# Antithrombotic Therapy in Elderly Patients with Acute Coronary Syndromes

**DOI:** 10.3390/jcm11113008

**Published:** 2022-05-26

**Authors:** Clara Bonanad, Francisca Esteve-Claramunt, Sergio García-Blas, Ana Ayesta, Pablo Díez-Villanueva, Jose-Ángel Pérez-Rivera, José Luis Ferreiro, Joaquim Cánoves, Francisco López-Fornás, Albert Ariza Solé, Sergio Raposerias, David Vivas, Regina Blanco, Daznia Bompart Berroterán, Alberto Cordero, Julio Núñez, Lorenzo Fácila, Iván J. Núñez-Gil, José Luis Górriz, Vicente Bodí, Manuel Martínez-Selles, Juan Miguel Ruiz Nodar, Francisco Javier Chorro

**Affiliations:** 1 Cardiology Department, Clinic University Hospital of Valencia, 46010 Valencia, Spain; sergiogarciablas@gmail.com (S.G.-B.); xcfemenia@gmail.com (J.C.); drlopezfornas@gmail.com (F.L.-F.); yulnunez@gmail.com (J.N.); vicente.bodi@uv.es (V.B.); francisco.j.chorro@uv.es (F.J.C.); 2 INCLIVA Biomedical Research Institute, 46010 Valencia, Spain; jlgorriz@gmail.com; 3 Department of Medicine, University of Valencia, 46010 Valencia, Spain; dazniabompart@gmail.com; 4 Department of Nursing, Faculty of Health Sciences, European University of Valencia, 46010 Valencia, Spain; francisca.esteve@universidadeuropea.es; 5 Centro de Investigación Biomédica en Red Enfermedades Cardiovasculares (CIBERCV), 28029 Madrid, Spain; jlferreiro@bellvitgehospital.cat (J.L.F.); acorderofort@gmail.com (A.C.); 6 Cardiology Department, Central University Hospital of Asturias, 33011 Oviedo, Spain; ana.ayestalopez@gmail.com; 7 Cardiology Department, La Princesa University Hospital, 28006 Madrid, Spain; pablo_diez_villanueva@hotmail.com; 8 Cardiology Department, University Hospital of Burgos, Isabel I University, 09003 Burgos, Spain; jangel.perezrivera@secardiologia.es (J.-Á.P.-R.); aariza@bellvitgehospital.cat (A.A.S.); 9 Cardiology Department, Bellvitge University Hospital, 08907 Barcelona, Spain; 10 Cardiology Department, Álvaro Cunqueiro University Hospital, 36213 Vigo, Spain; raposeiras26@hotmail.com; 11 Department of Medicine, Cardiovascular Institute, San Carlos University Hospital, Complutense University, 28040 Madrid, Spain; dvivas@secardiologia.es (D.V.); ibnsky@yahoo.es (I.J.N.-G.); 12 Cardiology Department, Quirónsalud Hospital of Valencia, 46010 Valencia, Spain; rb.delburgo@gmail.com; 13 Cardiology Department, San Juan University Hospital, 00921 Alicante, Spain; 14 Cardiology Department, General University Hospital of Valencia, 46014 Valencia, Spain; lfacila@gmail.com; 15 Department of Nephrology, Clinic University Hospital of Valencia, 46010 Valencia, Spain; 16 Cardiology Department, Hospital Universitario Gregorio Marañón, Universidad Europea, Complutense University, 28040 Madrid, Spain; mmselles@secardiologia.es; 17 Servicio de Cardiología, Hospital General Universitario de Alicante, Instituto de Investigación Sanitaria y Biomédica de Alicante ISABIAL, CIBER-CV, 03010 Alicante, Spain; ruiz_jmi@gva.es

**Keywords:** acute coronary syndrome, elderly, antiplatelet therapy, clopidogrel, prasugrel, ticagrelor

## Abstract

The treatment of acute coronary syndrome (ACS) in elderly patients continues to be a challenge because of the characteS.G.B.ristics of this population and the lack of data and specific recommendations. This review summarizes the current evidence about critical points of oral antithrombotic therapy in elderly patients. To this end, we discuss the peculiarities and differences reported referring to dual antiplatelet therapy (DAPT) in ACS management in elderly patients and what might be the best option considering these population characteristics. Furthermore, we analyze antithrombotic strategies in patients with atrial fibrillation (AF), with a particular focus on those cases that also present coronary artery disease (CAD). It is imperative to deepen our knowledge regarding the management of these challenging patients through real-world data and specifically designed geriatric studies to help resolve the questions remaining in their disease management.

## 1. Introduction

Cardiovascular disease constitutes one of the leading causes of death worldwide. The incidence of acute coronary syndromes (ACS) is especially high in the elderly, who constitute up to one-third of patients. Age also associates with an increased risk of recurrent ischemic events and death [1,2].

Antithrombotic therapy represents the main component of treatment in the setting of ACS. The focus is on antiplatelet therapy, but balancing the benefit in terms of reducing ischemic events with the bleeding risk is still complicated. In combination with aspirin, oral P2Y_12_ receptor inhibitors (clopidogrel, prasugrel, and ticagrelor) have been widely implemented as a first-line treatment strategy in patients with ACS and those undergoing percutaneous coronary intervention (PCI) [3]. However, the management of ACS in the elderly has turned out to be challenging, since compared with clopidogrel, prasugrel and ticagrelor involve an increased risk of bleeding, potentially offsetting their ischemic clinical benefit among more vulnerable patients [4].

Elderly patients with ACS usually present atypical characteristics, causing a delay in diagnosis and treatment. They require a multidimensional clinical approach, as they present multiorgan changes, frequent comorbidities, comedication, and reduced adherence to treatment, making them more vulnerable to pharmacological or interventional treatments [5,6]. These variables are associated with poorer outcomes, thus making it an even more significant challenge to select antiplatelet therapy in elderly patients [6].

Despite higher adverse events, including mortality, the older population is underrepresented in clinical trials, making it challenging to extrapolate recommendations to this cohort. This review summarizes the current evidence about some critical points of oral antithrombotic therapy in elderly patients.

## 2. Bleeding Risk in Elderly Patients with ACS and Antithrombotic Therapy

Current clinical practice guidelines [7,8,9] recommend dual antiplatelet therapy (DAPT) using a P2Y_12_ receptor inhibitor in combination with aspirin as a first-line treatment strategy in patients with ACS and patients undergoing PCI. Despite the exhaustive evidence of the clinical benefit of this therapy in terms of ischemic events, it poses a bleeding risk, which can be higher depending on the patient’s clinical characteristics.

The Clopidogrel in Unstable Angina to Prevent Recurrent Events (CURE) study was the first-ever trial to compare aspirin treatment alone with aspirin together with clopidogrel treatment in patients with ACS. This demonstrated the beneficial effects of the P2Y_12_ inhibitor. The clopidogrel group had significantly reduced risk of death from cardiovascular causes, myocardial infarction (MI) or stroke, and ischemic events. Furthermore, bleeding tended to be more common if coronary artery bypass grafting (CABG) was performed within five days of clopidogrel administration (8.5% with clopidogrel vs. 5.7% with placebo). The authors of CURE additionally performed a subanalysis including age subgroups (65 years old *n* = 6354 patients vs. >65 years old *n* = 6208 patients) that suggested that the rates and relative risks of the first primary outcome (death from cardiovascular causes, nonfatal MI, or stroke) were better with clopidogrel than with placebo for both subgroups. However, clopidogrel was associated with an increased risk of bleeding [10].

Ticagrelor and prasugrel are potent P2Y_12_ inhibitors that showed superiority over clopidogrel in the Platelet Inhibition and Patient Outcomes (PLATO) and in Trial to Assess Improvement in Therapeutic Outcomes by Optimizing Platelet Inhibition with Prasugrel Thrombolysis in Myocardial Infarction 38 (TRITON-TIMI 38) clinical trials. Both P2Y_12_ inhibitors significantly reduced rates of ischemic events, including stent thrombosis in patients with acute coronary syndromes with scheduled PCI, compared with clopidogrel. Nevertheless, both ticagrelor and prasugrel were associated with a slightly increased risk of major bleeding, including fatal bleeding [11,12].

The PEGASUS study included a total of 3083 patients ≥75 years (representing 15% of the total patients included in the trial). This randomized clinical trial showed that ticagrelor 60 mg was superior to placebo in reducing MACE (3 year KM rate of 11.02% vs. 13.5%, respectively). However, patients aged ≥75 years treated with ticagrelor 60 mg had a higher rate of major thrombolysis in myocardial infarction (TIMI) bleeding (3-year KM rate of 4.11% vs. 1.68% for ticagrelor and control, respectively). In addition, the study excluded all patients at high risk of bleeding, such as those with a previous history of stroke or a history of recent bleeding [13].

Despite the recommendations issued for the general population with non-ST-segment elevation acute coronary syndrome, data on optimal platelet inhibition in elderly patients is limited, so in the TRITON-TIMI 38 trial, the representation of elderly patients accounted for only 13%, whereas in the PLATO trial, 15% of the study population was >75 years old [12,14]. This constitutes one of the major problems regarding therapeutic decision making and assessment of bleeding risk in real-world situations.

Consequently, current guidelines do not make strong specific or differential recommendations regarding antiplatelet therapy for this high-risk subgroup, thus advising a balance between ischemic vs. bleeding risks [7]. Older patients might have multiple comorbidities such as diabetes mellitus, renal dysfunction, and anemia, all of which increase the risk of complications. In this scenario, the use of scores might be helpful to tailor antithrombotic treatment in order to maximize ischemic protection and minimize bleeding risk [15]. However, these scales do not consider variables associated with comorbidity or frailty, which could explain their loss of efficacy in elderly patients [16]. Although several tools are available, it is important to note that only a few have been validated for the older population [17] and that the potential use of different cutoff points cannot be ruled out, since it could be of help for accurately evaluating these complex patients [18].

### 2.1. Comparisons between Antiplatelet Therapies in Elderly Patients

#### 2.1.1. Clopidogrel vs. Prasugrel

In the TRITON-TIMI 38 trial, patients ≥ 75 years who were treated with prasugrel showed an increased risk of developing major bleeding (HR, 1.32; 95% CI, 1.03 to 1.68; *p* = 0.03) or fatal bleeding (0.4% vs. 0.1%; *p* = 0.002) compared with those treated with clopidogrel, resulting in a neutral net clinical benefit [12].

The usefulness of the 5 mg dose of prasugrel in elderly patients has been primarily evaluated in two clinical trials. Savonitto et al. compared a reduced dose of prasugrel (5 mg) vs. clopidogrel at the standard dose (75 mg) in 1443 patients >74 years with ACS undergoing PCI, using as primary end point a composite of mortality, MI, disabling stroke, and rehospitalization for cardiovascular causes or bleeding within one year. They observed that there were no differences in the primary endpoints between prasugrel (17.0% of occurrence) and clopidogrel (16.6% of occurrence) (HR, 1.007; 95% CI, 0.78–1.30; *p* = 0.955). However, the premature termination of the trial and the open-label design must be considered in the interpretation of the results [19]. In a secondary analysis of a randomized, double-blind clinical trial including 2083 patients 75 years of age or older, in which the authors also evaluated up to 30 months of treatment with prasugrel (5 mg) vs. clopidogrel (75 mg) in ACS patients without revascularization, prasugrel did not cause any increase in the rate of major bleeding in all age groups, including patients over 75 years of age [20].

Regarding the clinical strategy of platelet function monitoring to adjust therapy in low-risk patients undergoing elective coronary stenting, a French multicenter open-label randomized controlled superiority study (the ANTARTIC study) included patients aged 75 years or older who had undergone coronary stenting for acute coronary syndrome. The study aimed to assess the effect of platelet function monitoring with treatment adjustment in elderly patients stented for acute coronary syndrome. The primary endpoint (a composite of cardiovascular death, myocardial infarction, stroke, stent thrombosis, urgent revascularization, and Bleeding Academic Research Consortium-defined bleeding complications (types 2, 3, or 5) at 12 months follow-up) did not differ significantly between groups [21].

#### 2.1.2. Clopidogrel vs. Ticagrelor

In the prespecified subanalysis of the PLATO study comparing clinical outcomes in elderly (≥75 years of age) vs. younger (<75 years of age) patients, the authors concluded that ticagrelor reduced ischemia and mortality outcomes compared with clopidogrel without increasing the overall rate of major bleeding (17.2 vs. 18.3%; HR, 0.89; 95% CI, 0.74–1.08) [11], suggesting that the benefit demonstrated by ticagrelor could be extrapolated to the age group of older than 75 years.

Furthermore, in another subanalysis of the PLATO study in elderly patients, no significant difference was found in the benefit of ticagrelor over clopidogrel in patients ≥75 years compared with those <75 years in terms of major adverse cardiac events (MACE), MI, cardiovascular death, stent thrombosis, or all-cause mortality. Nevertheless, ticagrelor was more effective than clopidogrel in reducing MACE, and all-cause mortality across all age ranges included in the study, which was consistent with results published in PLATO [22]. In this study, 3237 patients with chronic kidney disease (CKD) (defined as CrCl level <60 mL/min) were analyzed whose median age was 74 years and of whom 46.3% were ≥75 years. In patients with CKD, ticagrelor significantly reduced the incidence of the primary endpoint (MACE) vs. clopidogrel, showing a more significant absolute risk reduction than that in patients with normal renal function. In patients with CKD, ticagrelor reduced total mortality compared with clopidogrel. The rates of major and fatal bleeding and major bleeding unrelated to CABG were not significantly different between the two randomized groups (CKD patients and patients with normal renal function) [22].

These results highlight that in elderly patients with high ischemic risk but low bleeding risk and in patients with renal failure (a very common comorbidity in elderly patients), ticagrelor could be a reasonable choice, although large-scale, head-to-head comparisons between ticagrelor and prasugrel in older patients are not currently available.

Supporting these findings, another study that analyzed data from the Bremen ST-elevation myocardial infarction (STEMI) registry that evaluated the impact of ticagrelor in patients older than 75 years reported that patients did not show an excess of bleeding events, and a reduction in the rates of major adverse cardiac or cerebrovascular events was observed compared with the rate in patients treated with clopidogrel (HR, 0.69; 95% CI, 0.49–0.97; *p* = 0.03), thus evidencing the safety and efficacy of ticagrelor in a real-world cohort of elderly patients with STEMI [23]. Similarly, the results of an analysis of two multinational registries (*n* = 16,653) evidence that ticagrelor did not show a significant increase in major bleedings compared with clopidogrel in older patients but significantly increased 1-year survival [24].

In discrepancy with these results, we identified the POPular AGE study [25]. This randomized clinical trial aimed to evaluate the efficacy and safety of clopidogrel (*n* = 500 patients) compared with ticagrelor or prasugrel (*n* = 502, 475 of whom (95%) received ticagrelor) in patients ≥70 years with ACS with a follow up of 12 months [26]. The results supported the use of clopidogrel in this population given the lower rate of bleeding compared with that in the group treated with ticagrelor (18% vs. 24%; HR 0.71, 95% CI 0.54 to 0.94; *p* = 0.02 for superiority) without resulting in a lower net clinical benefit or an increase in all-cause death, MI, stroke, or bleeding [25]. The high rates of medication discontinuation should be noted, especially in the ticagrelor group (47% in ticagrelor group vs. 22% in clopidogrel group), which could have generated a bias in the results.

Consistently with these last results, an observational analysis of the Swedish SWEDEHEART registry (*n* = 14,005) comparing the use of DAPT with clopidogrel (60.2%) or ticagrelor (39.8%) reported an increased risk of bleeding with the use of ticagrelor in elderly patients (≥80 years) with MI; however, the authors highlighted the need for a specific randomized clinical trial [27]. Further in this vein, in an observational study as part of the SCOPE study, which examined the safety of ticagrelor compared with clopidogrel in octogenarians with NSTE-ACS, the authors found that the rate of ischemic events was similar, while there was a significantly higher rate of bleeding complications with ticagrelor (2.2% vs. 7.1%; *p* = 0.009 and 0.7% vs. 5.5%; *p* = 0.04), so it should be used with caution in older patients with high bleeding risk [1].

#### 2.1.3. Prasugrel vs. Ticagrelor

The PRAGUE-18 study carried out a head-to-head comparison between ticagrelor and prasugrel in patients with acute myocardial infarction (mostly STEMI) undergoing primary PCI, including a subgroup of 121 patients (9.8%) aged ≥75 years. No significant differences were found in safety and efficacy between ticagrelor and prasugrel in the general population and the specific group aged ≥75 years. However, we should not overlook the limitations of the study, such as its open-label design, its premature termination due to futility, and the lack of power to draw a final conclusion [28].

In the absence of a specific study for the elderly population, the results of the Intracoronary Stenting and Antithrombotic Regimen Rapid Early Action for Coronary Treatment (ISAR-REACT) 5 clinical trial comparing ticagrelor vs. prasugrel in ACS patients are of interest. In the overall population of the study, a benefit of prasugrel, since this cohort of patients showed a lower incidence of death, MI or stroke, compared with ticagrelor was observed, whereas the incidence of major bleeding was not significantly different between the two groups [29]. A subanalysis of ISAR-REACT 5 suggested that a reduced dose of prasugrel (modified in elderly and low-weight patients) may still maintain the anti-ischemic effect while reducing the risk of bleeding vs. ticagrelor [30]. However, care should be taken interpreting these results because of the open-label design, high discontinuation rate, and exclusion of patients treated with prasugrel in the safety analysis. In fact, a heated debate in the scientific community has arisen regarding the external validity of the findings of the ISAR-REACT 5 trial and its impact on the ESC guidelines on NSTEACS [31]. Therefore, new studies must elucidate and validate the findings of this trial.

There is currently scarce use of the new P2Y_12_ inhibitors among elderly patients [17,32,33]. Although clinical practice guidelines recommend using ticagrelor or prasugrel as the first option in the overall population, there is an evident lack of data and representativeness in clinical trials in the elderly population. It should be taken into account that prasugrel, at 5 mg dose, should be used with caution in patients ≥75 years of age, as recommended in clinical practice guidelines, and that clopidogrel has not demonstrated superiority over prasugrel.

Table 1 provides a comparison of RCTs and the effect of oral P2Y_12_ inhibitor on the study population.

## 3. Duration of Dual Antiplatelet Therapy in Elderly Patients

After a percutaneous coronary intervention (PCI) with drug-eluting stents, there are competing risks of bleeding and thrombotic events in patients requiring antithrombotic therapies. In these situations, it is required to perform DAPT with aspirin and a P2Y_12_ receptor inhibitor to prevent thrombotic events [34]. The type and dosing regimen of antiplatelet agents in acute coronary syndrome is established and summarized in Table 2.

Many risk prediction tools have been recently developed to inform optimal decision making on DAPT duration after PCI [35]. The ESC guidelines endorse the use of risk scores to estimate the risk and benefits of different DAPT durations. Regarding the duration of DAPT, they recommend DAPT with a P2Y_12_ receptor inhibitor on top of aspirin for 12 months unless contraindicated (class of recommendation I, level of evidence A), although the possibility of shortening or lengthening the duration of DAPT is also contemplated according to the individual risk profile of each patient [7,9].

A meta-analysis including six randomized trials evaluated the optimal duration of DAPT (short duration of ≤6 months vs. long duration of 12 months) after drug-eluting stent implantation in elderly patients (defined, in this case, as ≥65 years). The study revealed the possible benefit of short-term DAPT in the elderly compared with people <65 years after implantation of new-generation DES due to a greater reduction in the risk of major bleeding [36].

Consistently with these results, the DAPT and PEGASUS-TIMI 54 studies showed that prolonged treatment with DAPT increased the risk of bleeding across all age subgroups [13,37]. Similarly, a population-based study [38] using the RENAMI registry, which included 12 European centers, compared long vs. short dual antiplatelet therapy in ACS patients treated with prasugrel or ticagrelor after coronary revascularization. Although only 185 (9.8%) out of the 1884 patients matched in the propensity score analysis were older than 75 years, the observed favorable effects of prolonged DAPT beyond 12 months on the reduction of MACE appeared to be diminished in older patients because of excess bleeds.

Other antithrombotic strategies have been developed with the intention to reduce the risk of hemorrhagic events, such as DAPT de-escalation or monotherapy with P2Y12 inhibitors [39,40]. However, a description of these strategies goes beyond the scope of this manuscript, and specific data on elderly patients regarding these regimens are still very scarce.

The clinical trials suggest that short-term DAPT treatment might be appropriate to avoid the increased risk of bleeding in elderly patients, especially those prone to hemorrhagic events. However, new evidence-based recommendations on the appropriate use and duration of DAPT in this cohort of patients are required, and further clinical trials are needed to support these recommendations. In fact, individualizing the duration of dual antithrombotic therapy in elderly patients remains reasonable.

## 4. Anticoagulation Therapy in Elderly Patients with Atrial Fibrillation

Atrial fibrillation (AF) is the most common sustained arrhythmia, and its incidence increases with age. Elderly patients are those with the highest risk of embolism events [41]. The comprehensive approach to AF patients currently focuses on two main anticoagulant treatment options, vitamin K antagonists (VKA) and direct oral anticoagulants (DOACs).

Even though the benefits of VKA have been demonstrated through extensive clinical experience, VKAs have several disadvantages, such as a narrow therapeutic window and variability of the coagulant effect, and regular controls of the International Index Normalized may limit its use in clinical practice. Thus, DOACs are the group of agents of choice in the elderly in the absence of contraindications [42,43]. Therapy with DOACs presents some advantages, such as the wide therapeutic window; it is not necessary to monitor anticoagulant activity, and they can be prescribed at fixed doses according to specific clinical characteristics [44].

Some clinical studies on anticoagulation in the AF population included a significant number of older patients. The RE-LY study included 7258 patients (40.1%) aged ≥75 years [45], the ROCKET AF study included 6229 patients (44%) aged ≥75 years with atrial fibrillation [46], and the ARISTOTLE trial included 31% of patients aged ≥75 years. The rates of stroke, all-cause death, and major bleeding were higher in the older age groups (*p* < 0.001 for all) [47]. Because of the higher risk at older age, these studies confirmed that DOACs showed superior or similar efficacy and safety with a lower rate of bleeding, especially intracranial, compared with VKA consistently across all age groups. Despite these efficacy data and the availability of more alternatives in the choice of drugs, anticoagulant treatment continues to be underused in patients with AF, especially in very elderly patients and in patients with disabilities, cognitive impairment, or high comorbidity. Moreover, there were relevant differences in the clinical profiles of patients aged 75 years or older included in these studies.

These studies present some specifications, such as the narrow follow-up, that may not always be applicable to real-life situations, highlighting the need to conduct real-life studies. An updated meta-analysis, including 27 studies, clinical trials, and cohort studies, concluded that apixaban (HR, 0.66; 95%CI, 0.55–0.80) and dabigatran (HR, 0.83; 95%CI, 0.70–0.97) significantly reduced the major bleeding risk vs. warfarin. Furthermore, apixaban (HR, 0.56; 95%CI, 0.42–0.73), dabigatran (HR, 0.45; 95%CI, 0.39–0.51), and rivaroxaban (HR, 0.66; 95%CI, 0.49–0.88) significantly reduced the risk of intracranial bleeding vs. warfarin. The finding suggested that reduced doses of direct oral anticoagulants were associated with a slightly better safety profile but with a marked reduction in stroke prevention effectiveness [48].

In recent years, several studies have been published analyzing the use of DOACs in routine clinical practice [49,50,51,52,53,54]. In general, in elderly patients with nonvalvular AF, DOACs were effective and safe and showed superiority over VKA in elderly patients in terms of stroke or bleeding risk prevention.

Nevertheless, some studies found that there was a tendency to use inadequate doses in elderly patients, generally due to underdosing, leading to inadequate protection against thromboembolic events and even increased mortality compared with correct anticoagulation. Thus, it is estimated that in the elderly population, approximately 30–40% of patients received inadequate doses of DOACs [55,56,57,58,59,60].

On the other hand, antiplatelet therapy presents low efficacy for preventing stroke, and therefore, its use for this indication is not justified. The evidence about the effectiveness of antiplatelet agents for stroke prevention in AF is minimal, and its use is not recommended [61].

## 5. Patients with AF and Coronary Artery Disease

Current guidelines for AF recommend that most patients with AF and patients with chronic CAD without events in 1 year should receive monotherapy with DOACs, which are considered a safe and effective standard therapy for long-term management [7,62]. As per the EHRA guidelines, they should be considered only as an additional antiplatelet agent in individual patients at very high ischemic risk and a low bleeding risk [62].

Until recently, there were only indirect data from the pivotal phase 3 trials using DOACs and some observational data on whether it might be safe to transition to DOAC monotherapy in patients with CAD. In elderly patients, concomitant use of DOACs with strong platelet inhibitors (prasugrel, ticagrelor), dual platelet inhibition, or nonsteroidal anti-inflammatory drugs (NSAIDs) should be restricted to the minimal duration considered crucial in order to prevent ischemic events. Concomitant antiplatelet drugs appeared to increase the risk for significant bleeding in RE-LY without affecting the advantages of dabigatran over warfarin. Patients with high coronary risk, such as elderly patients, may be at risk for perioperative cardiovascular events during DOAC interruption due to the absence of antithrombotic therapy [62].

A meta-analysis that included six trials (a total of 8855 patients with nonvalvular AF and stable CAD but generally including patients at low ischemic risk) compared DOAC monotherapy vs. DOAC plus single antiplatelet therapy. It showed that DOAC monotherapy provided more efficacy than DOAC plus single antiplatelet therapy with lower bleeding risk. There was no significant difference in MACE in AF patients treated using DOAC plus single antiplatelet therapy compared with those treated with DOAC monotherapy (HR 1.09; 95% CI, 0.92–1.29). On the other hand, DOAC plus antiplatelet therapy was associated with a significantly higher risk of major bleeding compared with DOAC monotherapy (HR 1.61; 95% CI, 1.38–1.87), as well as higher risk of net adverse events (NAE) (HR, 1.21; 95% CI, 1.02–1.43). Although these results were the main data we found in this scenario, we must take into account the methodological limitations of the AFIRE study and the premature stopping of the trial due to an increase in mortality in the combination therapy arm in the interpretation of the results [63,64].

These results support that DOAC alone may confer the same benefits with fewer risks in patients without a high risk of ischemic events, suggesting that there is a large subgroup of patients with stable CAD for whom antiplatelet therapy should not be prescribed as a preventive medication. However, an individualized approach is still mandatory when deciding the optimal combination and duration of antithrombotic agents.

## 6. Dual Antiplatelet Therapy (DAPT) Strategy in Elderly ACS Patients Undergoing Coronary Artery Bypass Grafting (CABG)

Coronary artery bypass graft surgery (CABG) is an effective treatment for patients with ischemic heart disease but presents a high risk of occlusion after bypass surgery. By ten years after surgery, the majority of vein grafts—main used tube—either occlude or develop a heavy burden of atherosclerosis, leading the patients who have undergone CABG to a subsequent high risk of an ischemic event, including myocardial infarction (MI), stroke, or death [65,66]. However, in the elderly, CABG seems to be more beneficial than PCI in terms of survival [67] and development of an MI or stroke or subsequent revascularization [68].

Even if guidelines recommend DAPT after CABG for patients with ACS, the evidence for these recommendations is limited [9,69,70], a fortiori in elderly patients, in whom DAPT use is suboptimal for patients undergoing CABG [71]. There are several gaps in the current evidence about DAPT in elderly ACS patients undergoing CABG, particularly whether DAPT should be started after CABG, when the postoperative DAPT should restart, and the optimal point of DAPT discontinuation [9]. Indeed, the main concern of this therapy in a CABG context is about the perioperative bleeding risk, given that continuing DAPT until CABG highly increases this risk [9]. The American College of Cardiology (ACC), the American Heart Association (AHA), and the European Society of Cardiology (ESC) recommend specific interruption and resumption of DATP on a patient undergoing CABG [9,72]. The Society of Thoracic Surgeons (STS) recommends that precisely restarting the DAPT drug as soon as the bleeding risk is diminished may have a secondary benefit of increasing early vein graft patency [73]. The STS also suggests that it could be more relevant for patients already on DAPT to make decisions about surgical timing based on platelet inhibitions tests rather than applying a prespecified period of surgical delay [74]. Nevertheless, these recommendations should be individualized for the elderly population, a particular category of patients with higher bleeding risk [22].

## 7. Conclusions

The clinical management of ACS in the elderly continues to be a challenge for healthcare professionals involved in its diagnosis and treatment. The inherent complexity of this patient profile with comorbidities and geriatric syndromes requires further studies and issuance of specific recommendations to ensure the greatest clinical benefit can be achieved in these patients.

Several questions regarding the optimal antithrombotic therapy in elderly patients with ACS, AF, or the combination of AF and CAD remain unanswered. Indeed, a thorough assessment of the balance among ischemic, thromboembolic, and hemorrhagic risks is mandatory. Furthermore, an individualized approach on a case-to-case basis is crucial in order to decide the optimal antithrombotic strategy and its duration for each patient.

Real-world data are still lacking, and further studies with geriatric assessment should be considered to achieve a holistic approach and optimize treatment based on the underlying age-related vulnerability and frailty in the elderly, which should be taken into account to achieve their optimal management.

## Figures and Tables

**Table 1:** Comparison of RCTs for oral P2y12 Inhibitors [9,25,30].

Trial Name and Design	Inclusion Criteria	Study Arms	Efficacy Endpoint	Safety Endpoint	Interpretation
CURE Clopidogrel vs. Placebo (double-blind, randomized)	Age >21 ACS without STEMI (suspected UA or NSTEMI) Presentation <24 h after onset of symptoms	Clopidogrel 300 mg × 1, then 75 mg + ASA 75–325 mg/d (*n* = 6259) Placebo + ASA 75–325 mg/d (*n* = 6303)	Event (at end of the study) Death from CV causes Nonfatal MI Stroke Occurrence (*p* < 0.001) Clopidogrel: 9.3% Placebo: 11.4%	Event (at end of the study) Major bleeding Occurrence (*p* = 0.001) Clopidogrel: 3.7% Placebo: 2.7%	Clopidogrel reduced the efficacy endpoint by 20%
PLATO Clopidogrel vs. Ticagrelor (double-blind, randomized)	ACS with or without ST-segment elevation Onset of the symptoms within the previous 24 h	Clopidogrel 300 mg × 1, then 75 mg/d + ASA 75–100 mg/d (*n* = 9291) Ticagrelor 180 mg × 1, then 90 mg twice/d + ASA 75–100 mg/d (*n* = 9333)	Event (at month 12) Death from vascular causes MI Stroke Occurrence (*p* < 0.001) Clopidogrel: 11.7% Ticagrelor: 9.8%	Event (at month 12) PLATO-defined major bleeding Occurrence (*p* = 0.43) Clopidogrel: 11.2% Ticagrelor: 11.6%	Ticagrelor reduced the efficacy endpoint by 16% compared with Clopidogrel
TRITON-TIMI 38 Clopidogrel vs. Prasugrel (double-blind, randomized	Planned PCI for ACS	Clopidogrel 300 mg × 1, then 75 mg/d + ASA 75–100 mg/d (*n* = 6795) Prasugrel 60 mg × 1, then 10 mg /d + ASA 75–100 mg/d (*n* = 6813)	Event (at month 15) Death from CV causes Nonfatal MI Nonfatal stroke Occurrence (*p* < 0.001) Clopidogrel: 12.1% Prasugrel: 9.9%	Event (at month 15) Non-CABG-related TIMI major bleeding Occurrence (*p* = 0.43) Clopidogrel: 1.8% Prasugrel: 2.4%	Prasugrel reduced the efficacy endpoint by 19% compared with Clopidogrel
POPular Age Study Clopidogrel vs. Ticagrelor or Prasugrel (open label, randomized)	Age >70 NSTE-ACS	Clopidogrel 300 or 600 mg × 1, then 75 mg (*n* = 500) Ticagrelor 180 mg × 1, then 90 mg twice/d (*n* = 475) OR Prasugrel 60 mg × 1, then 10 mg /d (*n* = 27)	Event All-cause death MI Stroke	Event Major bleeding Occurrence (*p* = 0.02) Clopidogrel: 18% Ticagrelor: 24%	Clopidogrel led to a lower rate of bleeding in the elderly population compared with Ticagrelor
ISAR-REACT Ticagrelor vs. Prasugrel (multicentric, open label, randomized)	ACS (STEMI, NSTEMI, UA) Planned coronary angiography	Ticagrelor 180 mg × 1, then 90 mg twice/d (*n* = 2012) Prasugrel 60 mg × 1, then 10 mg /d OR 5 mg/d if > 75 years old (*n* = 2006)	Event (at month 12) All-cause death MI Stroke Occurrence (*p* = 0.006) Ticagrelor: 14.6%% Prasugrel: 12.7%	Event (at month 12) BARC-defined bleeding complications Occurrence (*p* = 0.46) Ticagrelor: 10.6% Prasugrel: 8.1%	Lower dose of Prasugrel in case of elderly population maintained anti-ischemic efficacy and protected against excess risk of bleeding

ACS, acute coronary syndrome; UA, unstable angina; STEMI, ST-segment elevation myocardial infarction; NSTEMI, non-ST-elevation myocardial infarction; ASA, acetylsalicylic acid; CV, cardiovascular; MI, myocardial infarction; PLATO, Platelet Inhibition and Patient Outcomes; PCI, percutaneous coronary intervention; CABG, coronary artery bypass graft surgery; TIMI, thrombolysis in myocardial infarction; NSTE, non-ST-elevation; BARC, Bleeding Academic Research Consortium.

**Table 2:** Type and dosing regimen of antiplatelet agents in acute coronary syndrome.

Antiplatelet Drugs	
Salicylates	P2Y_12_ Receptor Inhibitors (Oral or Intravenous)
Aspirin - LD of 150–300 mg orally or - LD of 75–250 mg intravenous if oral ingestion is not possible followed by oral MD of 75–100 mg once daily.	Clopidogrel LD of 300–600 mg orally, followed by an MD of 75 mg once daily. No specific dose adjustment in CKD patients. - Contraindications: ○ Hypersensitivity to the active substance or to any of the excipients. ○ Active pathological bleeding, such as peptic ulcer or intracranial hemorrhage. ○ Severe hepatic insufficiency.
	Prasugrel In patient aged ≥75 years, use prasugrel with caution if treatment is deemed necessary: LD of 60 mg orally followed by an MD of 5 mg once daily. In patients with body weight <60 kg, an MD of 5 mg once daily is recommended. No specific dose adjustment in CKD patients. Prior stroke is a contraindication for prasugrel. - Contraindications: ○ Hypersensitivity to the active substance or to any of the excipients. ○ Active pathological bleeding. ○ Severe hepatic insufficiency (Class C on the Child–Pugh scale). ○ History of stroke or transient ischemic attack (TIA).
	Ticagrelor LD of 180 mg orally, followed by an MD of 90 mg twice a day. No specific dose adjustment in CKD patients. - Contraindications: ○ Hypersensitivity to the active substance or to any of the excipients. ○ Active pathological bleeding. ○ History of intracranial hemorrhage. ○ Severe hepatic impairment. ○ Concomitant administration of ticagrelor with potent CYP3A4 inhibitors.

CKD = chronic kidney disease; MD = maintenance dose; LD = loading dose. Note: table adapted from [7].

## Data Availability

Not applicable.
